# The Influence of Episodic Future Thinking and Graphic Warning Labels on Delay Discounting and Cigarette Demand

**DOI:** 10.3390/ijerph182312637

**Published:** 2021-11-30

**Authors:** Gideon P. Naudé, Sean B. Dolan, Justin C. Strickland, Meredith S. Berry, David J. Cox, Matthew W. Johnson

**Affiliations:** Department of Psychiatry and Behavioral Sciences, School of Medicine, Johns Hopkins University, Baltimore, MD 21224, USA; sdolan8@jhmi.edu (S.B.D.); jstric14@jhmi.edu (J.C.S.); mberry@ufl.edu (M.S.B.); dcox@endicott.edu (D.J.C.); mwj@jhu.edu (M.W.J.)

**Keywords:** episodic future thinking, graphic warning labels, cigarette smoking, behavioral economics, delay discounting, demand

## Abstract

Delay discounting and operant demand are two behavioral economic constructs that tend to covary, by degree, with cigarette smoking status. Given historically robust associations between adverse health outcomes of smoking, a strong preference for immediate reinforcement (measured with delay discounting), and excessive motivation to smoke cigarettes (measured with operant demand), researchers have made numerous attempts to attenuate the extent to which behaviors corresponding to these constructs acutely appear in smokers. One approach is episodic future thinking, which can reportedly increase the impact of future events on present decision making as well as reduce the reinforcing value of cigarettes. Graphic cigarette pack warning labels may also reduce smoking by increased future orientation. Experiment 1 evaluated the combined effects of episodic future thinking and graphic warning labels on delay discounting; Experiment 2 evaluated solely the effects of episodic future thinking on delay discounting and operant demand. We observed no statistically significant effects of episodic future thinking when combined with graphic warning labels or when assessed on its own. These results serve as a call for further research on the boundary conditions of experimental techniques reported to alter behaviors associated with cigarette smoking.

## 1. Introduction

Factors contributing to harmful choices have been extensively studied using behavioral economics, a discipline that integrates principles of psychology and microeconomics. An account of substance use rooted in behavioral economic theory, known as the reinforcer pathologies model [1,2], theorizes that cigarette smoking and other drug taking behaviors occur within a microeconomic framework where (1) preference for immediate reinforcement despite suboptimal long-term outcomes and (2) excessively high motivation to consume cigarettes (and/or other substances) interact, manifesting as an extended pattern of chronically unhealthy behavior [3,4].

The first of these mechanisms, delay discounting, is perhaps the most widely researched behavioral economic construct and refers to the subjective devaluation of an outcome as a function of delay to occurrence.

There is extensive evidence linking greater delay discounting to those with substance use disorders, with ties to cigarette smoking among the most robust [5,6]. According to the *Diagnostic and Statistical Manual of Mental Disorders* [7], people who consume harmful substances like cigarettes may experience a recurring cycle involving a desire to inhibit unhealthy choices, failure to inhibit these choices, and subsequent self-disappointment and regret [8]. Specifically, cigarette smokers may desire better physical health and personal appearance while nevertheless continuing to choose the immediate rewards produced by smoking.

The second mechanism of the reinforcer pathologies model, operant demand, provides a quantitative characterization of the extent to which one will continue consuming a commodity amidst a range of constraints or prices [9,10]. Behavioral pharmacologists have extensively employed operant frameworks in laboratory settings to understand value and motivation associated with drug reinforcement [11,12]. Over the last 20 years, simulation tasks that model demand for cigarettes and other abused substances [13,14] have increasingly been used as an alternative to laboratory self-administration procedures and typically require far less time and resources to administer. Importantly, there is accumulating evidence suggesting that reinforcer value modeled using hypothetical outcomes corresponds to actual consumption [15,16,17] and converges with measures of substance use severity [18,19].

Given greater delay discounting and demand are associated with addiction, there have been numerous experimental attempts to alter these behavioral processes [20,21,22]. Episodic future thinking (EFT) is one such method and involves directing attention to personally relevant future events as a means of “expanding one’s temporal window” and increasing the likelihood delayed consequences will factor into the value of immediate reinforcement [23,24,25]. Stein et al. [26,27] found EFT manipulations were moderately associated with lower rates of monetary delay discounting and less demand for cigarettes as measured through self-administration and simulated purchasing. Indeed, recent reviews and meta-analyses highlight EFT as a generally reliable means of altering decisions involving an intrinsic tradeoff with the future [22,28].

Another method that may influence intertemporal decisions among cigarette smokers involves directing attention to graphic images of high-probability smoking-related illnesses when individuals regularly smoke cigarettes. For example, evidence suggests that incorporating graphic warning labels (GWLs) on packs of cigarettes in behavioral economic decision tasks can reduce the reinforcing value of cigarettes, presumably by increasing the salience of delayed outcomes [29]. There is also recent research linking perceived effectiveness of cigarette warning labels to lower rates of delay discounting of monetary rewards [30]. It thereby follows that GWLs may implicitly invoke EFT similarly to other approaches associated with lower rates of delay discounting (e.g., the effects of explicit-zero framing on delay discounting of monetary gains and losses [31]). By bringing attention to future consequences, GWLs and these other methods may be conceptualized as expanding one’s temporal window into the future, thereby increasing the degree to which delayed consequences may mitigate the reinforcing value of immediate rewards.

The present experiments were conducted to reproduce and extend previously reported behavioral economic findings involving EFT in cigarette smokers. In Experiment 1, we examined the combined roles of EFT and GWLs on monetary delay discounting, and in Experiment 2, we sought to examine the distinct role of EFT on delay discounting and demand for cigarettes. As clinical researchers strive to bring experimental techniques such as EFT closer to the point of application, it is important to test the robustness of these approaches and examine possible boundary conditions of their effectiveness.

## 2. Experiment 1

The primary aim of Experiment 1 was to examine the combinative effects of EFT and GWLs on delay discounting. We speculated that GWLs may implicitly invoke EFT and hence it was possible that using these approaches in combination would have greater effects on discounting than use of EFT alone. We therefore sought to examine monetary delay discounting as a function of EFT and GWLs using a factorial design in an online crowdsourced sample of adult cigarette smokers.

### 2.1. Method

#### 2.1.1. Participant Sample

Two hundred and ten cigarette smokers (*M*_age_ = 36.98 years; *SD* = 10.12) were recruited from Amazon Mechanical Turk (mTurk) and received monetary compensation for their participation in these online experimental tasks. To be eligible, participants needed to be cigarette smokers (i.e., answer “Yes” to the question “Do you smoke cigarettes?”) and have their work approved by mTurk requesters in no less than 95% of cases. In addition, participants needed to endorse using one of 11 cigarette brands that were presented as pictures of the cigarette pack: Marlboro (“Reds”, “Golds”, Menthol), Camel (“Blue”, “Filters”, Menthol), Newport (Menthol, Non-menthol), or Pall Mall (“Blue”, “Red”, Menthol). Cigarette brands were used as an inclusion criterion to present participants’ usual brand cigarette packages with or without the GWL, and these brands were chosen as they comprise the majority of cigarette sales in the United States [32,33]. Participation was voluntary, anonymous, and included $1 for completing the study and a $2 bonus for following instructions and passing attention checks. The sample was predominantly White (79%; *n* = 166) and approximately half identified as female (48%; *n* = 101). Participants reported smoking a mean of 14 cigarettes per day (*SD* = 8).

#### 2.1.2. Experimental Conditions

Participants were randomly assigned to 1 of the following 4 conditions: (1) EFT with regular cigarette packs; (2) EFT with GWL packs; (3) episodic recent thinking (ERT) with regular cigarette packs; and (4) ERT with GWL packs.

##### Episodic Events

Participants assigned to one of the EFT conditions generated three individualized future events they were looking forward to and could vividly imagine, providing specific details about where they would be, what they would be doing, who they would be with, and how they would feel. Participants then typed a one-sentence summary of each event into an entry field to be textually displayed on the screen during each choice in the discounting tasks (see Supplementary Materials). Time frames associated with these events were 1 month (displayed during delays 1 and 2), 1 year (displayed during delays 3 and 4), and 10 years (displayed during delays 5–7). Participants assigned to one of the ERT conditions were asked to remember and provide positive events that happened 1 day, 3 days, and 10 days ago that were displayed across delays in the same manner as the EFT conditions.

##### Cigarette Pack Labels

Participants viewed packs of their usual brand of cigarettes either featuring images of acutely diseased lungs and the words “WARNING: Cigarettes cause fatal lung disease” [29] or with the regular brand packaging. The respective image appeared on the screen while participants were asked to think about their episodic events (described above) and instructions specified they could not advance to the next page until 1 min had elapsed (see Supplementary Materials). Additional measures related to participants’ GWL response were collected, however, we do not report them here.

### 2.2. Delay Discounting Task

Participants completed a delay discounting task using a visual analog scale (VAS; [34]) and were asked to estimate the amount of immediately available money that would be equivalent to receiving $100 after the following delays: 1 day, 1 week, 1 month, 6 months, 1 year, 5 years, and 10 years. Instructions read:

“Imagine the following hypothetical (pretend) scenario:

You are presented with a choice between money now or later. For the money now option, the money is deposited automatically into your bank account immediately. For the money later option, the money is automatically deposited into your bank account after the specified time.

Your job is to use the slider tool to tell us the amount of money that you would like to receive immediately that would make you feel JUST AS GOOD as you would if you were to receive money after the specified time. Although the scenarios are pretend, we ask that you consider each scenario as if it was real and as if it was the only scenario you would face today. Finally, when considering each scenario, you should take into account your financial circumstances (e.g., current account balance, rent or bills due)”.

### 2.3. Tobacco Use Screening Measures

#### 2.3.1. Fagerström Test for Cigarette Dependence (FTCD)

The FTCD [35,36] is a widely used instrument measuring cigarette dependence, where scores (0–10) are described by the categories of low dependence (0–2), low-to-moderate dependence (3–4), moderate dependence (5–7), and high dependence (≥8).

#### 2.3.2. Contemplation Ladder

We assessed participants’ intention to quit smoking using the Contemplation Ladder [37] which measures the extent to which a person is ready to consider giving up smoking. Participants received the following instructions: “Each rung on this ladder represents where various smokers are in their thinking about quitting. Please select the number that indicates where you are now”. The scale ranged from 0 (no intention to quit) to 10 (currently taking action to quit).

### 2.4. Data Analysis

Delay discounting data were screened for systematic responding using criteria published previously [38], where (1) indifference points exceeding the previous indifference point by more than 20% of the larger-delayed amount and (2) cases where the final indifference point was not less than the first by more than 10% of the larger-delayed amount were omitted listwise. As a point estimate of delay discounting, we calculated the area under the discounting curve (AUC [39]). To conform to distributional assumptions of parametric statistical tests, AUC values were square root transformed prior to analysis. For demographic comparisons, categorical variables were analyzed using Pearson chi-squared tests, ordinal variables using Kruskal–Wallis tests, and continuous variables using one-way ANOVAs. Bivariate associations are reported for descriptive purposes using Spearman correlations. Finally, between-group comparisons of AUC (where lower values indicate greater discounting) were conducted using a 2 × 2 ANOVA. Statistical tests (across Experiments 1 and 2) were performed using SPSS version 27 and GraphPad Prism version 9.

### 2.5. Results

#### 2.5.1. Sample Characteristics

Forty-seven participants failed at least one of the embedded attention checks and were omitted listwise. An additional two cases were omitted as a result of entering nonsensical text into the episodic event entry fields. A total of 56 participants violated at least 1 of the criteria for systematic discounting (criterion 1 only: *n* = 14 cases; criterion 2 only: *n* = 25; both criteria: *n* = 17), leaving a final sample of *n* = 105 (EFT: *n* = 25; ERT: *n* = 28; EFT_GWL_: *n* = 24; ERT_GWL_: *n* = 28). Of note, previous reports [26] speculated that instances of cases flagged for failing criterion 2 of Johnson and Bickel’s [38] algorithm (i.e., largely invariant preference for the delayed option) may have been due to the EFT manipulation; however, we found no differences in the number of otherwise systematic cases flagged under this criterion across the four groups, χ^2^(3, *N* = 130) = 1.37, *p* = 0.71.

Randomly assigned groups did not differ significantly with respect to age (*F*_3,101_ = 1.51, *p* = 0.22), sex (χ^2^_3, *N*_ *_=_*_105_ = 1.39, *p* = 0.71), race (χ^2^_3, *N*_ *_=_*_105_ = 4.40, *p* = 0.22), level of education (χ^2^_3_ = 1.59, *p* = 0.66), income (χ^2^_3_ = 2.15, *p* = 0.54), cigarettes smoked per day (*F*_3,101_ = 1.61, *p* = 0.19), scores on the FTCD (*F*_3,101_ = 1.00, *p* = 0.40), or intentions to quit smoking (Contemplation Ladder; *F*_3,101_ = 0.75, *p* = 0.53). See Table 1 for group summary statistics.

#### 2.5.2. Bivariate Associations

Spearman rank correlations between sample demographics, smoking variables, and delay discounting are reported in Table 2, a subset of which we report here. There were significant positive associations between participant age and cigarettes smoked per day (*r*_s_ = 0.40, *p* < 0.001) as well as scores on the FTCD (*r*_s_ = 0.27, *p* = 0.01). Level of education was inversely associated with cigarettes smoked per day (*r*_s_ = −0.26, *p* = 0.01) and scores on the FTCD (*r*_s_ = −0.22, *p* = 0.03), the latter of which was inversely associated with reported intentions of quitting smoking (*r*_s_ = −0.26, *p* = 0.01). Finally, we observed a positive association between annual income and delay discounting (*r*_s_ = 0.21, *p* = 0.03), where higher income was associated with higher AUC (less discounting).

#### 2.5.3. Delay Discounting

**Figure 1:**
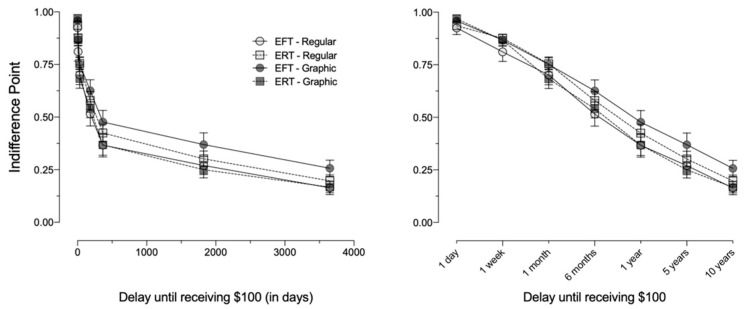
Mean indifference points (±*SEM*) on the delay discounting task for Experiment 1. EFT—episodic future thinking; ERT—episodic recent thinking. Left panel: delays presented on a linear *x*-axis to depict the shape of the discounting function. Right panel: delays presented on an *x*-axis with intervals equidistantly spaced to facilitate examination of all values.

**Figure 2:**
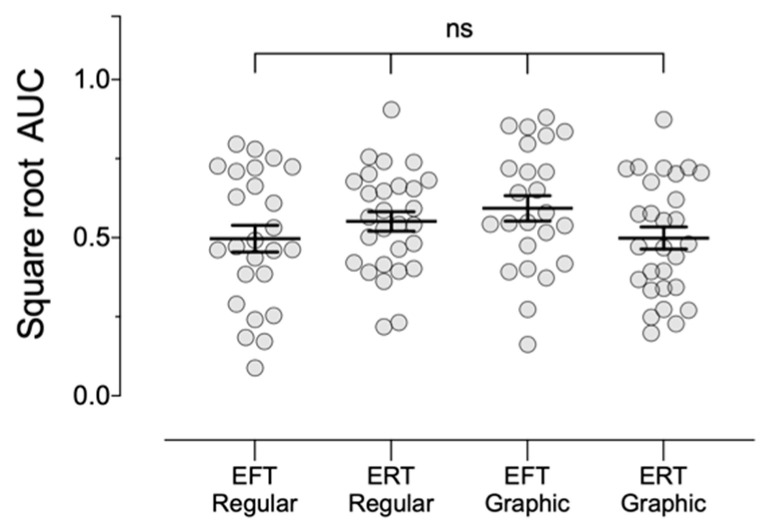
Mean square root transformed area under the curve (AUC) values (±*SEM*) for Experiment 1. EFT—episodic future thinking; ERT—episodic recent thinking. Regular—commercial cigarette packs; Graphic—commercial packs with graphic warning label. *Note*. Although no significant differences emerged in the omnibus and pairwise tests, GWLs resulted in slightly less discounting in the EFT conditions and slightly more discounting in the ERT conditions, resulting in a significant cross-over interaction (see text for statistical comparisons).

Figure 1 and Figure 2 depict the mean indifference points and AUC, respectively, for each of the four groups. We observed no statistically significant differences in delay discounting as a function of the EFT (*F*_1,101_ = 0.28, *p* = 0.60, $\eta_{p}^{2}$ = 0.003) or GWL manipulations, (*F*(1,101) = 0.35, *p* = 0.56,$\;\eta_{p}^{2}$ = 0.003). There was, however, a significant EFT × GWL cross-over interaction (*F*_101_ = 4.04, *p* = 0.047, $\eta_{p}^{2}$ = 0.04), where GWLs were associated with less discounting when paired with EFT and greater discounting when paired with ERT.

### 2.6. Conclusions

In the present experiment, neither individual nor combined EFT and GWL manipulations were associated with significant differences in monetary delay discounting. Given previous research showing a moderate effect of EFT in decreasing monetary delay discounting [26,27], we hypothesized the combinative effects of EFT and GWLs (as evidenced by the significant cross-over interaction) may have obscured the distinct roles of each stimulus presentation. After failing to reproduce previous EFT findings using a factorial design, we attempted to replicate those previous findings more directly in Experiment 2. In addition to comparing the effects of EFT on monetary delay discounting, we examined the effects of EFT on demand for cigarettes using a simulated purchasing task, as well as the degree to which participants could guess whether they were assigned to the experimental or control groups [27].

## 3. Experiment 2

### 3.1. Method

#### 3.1.1. Participant Sample

A new mTurk sample of 412 cigarette smokers (*M*_age_ = 35.73 years; *SD* = 9.94) completed Experiment 2. Eligibility criteria and compensation were identical to that of Experiment 1. The sample was largely White (76%; *n* = 312) and approximately sex-balanced (48% females; *n* = 196). The average number of cigarettes smoked per day was 14 (*SD* = 9) and comparable to what participants reported in Experiment 1.

#### 3.1.2. Measures

Participants were randomly assigned to either EFT or ERT conditions and generated the respective episodic events according to procedures outlined in Experiment 1. In addition to the discounting task, participants completed a cigarette purchase task (CPT), reporting the number of hypothetical cigarettes they would purchase and consume at various prices over a 24 h period. Cigarette purchasing was assessed across the following escalating price points: $0.01, $0.03, $0.06, $0.10, $0.30, $0.60, $1, $3, $6, $10, $30, $60, $100. Instructions read:

“The following questions are hypothetical (pretend), but please answer as though the consequences were real. This means you should take into account your current financial situation and any other factors about your current life circumstances when answering.

Imagine that you have finished the study and will spend the next day in your usual home environment. Additionally, imagine that you have the chance to buy your usual brand of cigarettes for your own personal use within the next day (24 h). You can buy as many cigarettes as you like, but you cannot sell, trade, or give them away, and you cannot save them for more than a day. Other than the fact that the cigarettes are for your own use within the next day, there is no limit to the number of cigarettes you can buy. Please do not buy more than you will use.

The following questions will ask you how many of your usual brand of cigarettes you would buy if they were sold at various prices. For each question, enter the number of cigarettes you would buy, and enter zero if you would not buy any at that price. Please consider each of the questions separately, meaning that if you buy cigarettes in one question, pretend that you do not have them when you answer the other questions. In other words, when you are answering each question, pretend that it is the only question being asked of you today”.

Prices were presented one at a time with the most distal self-generated episodic events (i.e., 10 years from now or 10 days ago for the EFT and ERT groups, respectively) displayed on the screen at each price (cf. [26,27]).

#### 3.1.3. Demand Characteristics

The survey concluded with a question asking participants to rate their level of confidence that they were in the experimental group [27]. Participants read the following: “Experimental studies will often assign participants to either an ‘‘experimental’’ group that receives active treatment or a ‘‘control’’ group that does not receive the active treatment. Outcomes from these two groups can then be compared to each other to understand the effects of the active treatment. Please use the scale below to rate your level of confidence that you were assigned to the ‘‘experimental’’ group in this study (i.e., the one that received active treatment)”.

The scale ranged from 0 (“I definitely WAS NOT assigned to this group”) to 100 (“I definitely WAS assigned to this group”). Previous research suggests that participants assigned to the experimental groups were no better than controls in discerning whether they were in the experimental or control group [27].

### 3.2. Data Analysis

Methods of screening for attention and systematic discounting were the same as those used in Experiment 1. Area under the curve was again used as the summary measure of discounting and the resulting values were square root transformed prior to statistical analysis. Orderliness of purchase task data was evaluated using previously published criteria [40,41]. Cases were considered nonsystematic if (1) consumption at one price was greater than consumption at the previous price by more than 20% and (2) consumption greater than 0 was reported after endorsing 0 consumption at a lower price.

Responses on the CPT were analyzed using the exponential model of demand [42]:

log *Q* = log *Q*_0_ + *k* (*e*^−α(*Q*^_0_^• *C*)^ − 1) (1)

where *Q* is consumption at each price (i.e., *C*) and *Q*_0_ is consumption when cost is minimal. In the present experiment, the number of hypothetical cigarettes purchased at $0.01 was used as *Q*_0_ (hereafter referred to as intensity of demand) rather than the model-derived index. The parameter *k* is the range of group consumption in logarithmic units (calculated as the difference of the logarithms of the mean maximum and mean minimum consumption values; *k* = 2.49 in the present experiment), and α is the rate of change in elasticity across the demand curve. To plot these data in double-logarithmic space, the first instance of “0” consumption for each participant was replaced with 0.10, with subsequent “0”s at higher prices not analyzed. Important to note is that participants reporting 0 or invariant consumption across prices nevertheless provide valuable data and as such were omitted only from analyses of the α parameter, as these response patterns result in an undefined parameter space when nonlinear curve fitting. In addition, intensity values were examined for outliers using the ROUT method [43] with false discovery rate (Q) set to 1%, resulting in 18 cases flagged (8 of these cases yielded otherwise systematic data and were therefore omitted only from demand analyses). Values of intensity and α were square root and natural log (ln) transformed, respectively, prior to statistical analysis. Categorical demographic analyses were conducted using Pearson chi-squared tests, while ordinal and continuous pairwise analyses were conducted using Mann–Whitney *U* tests and independent-samples *t* tests, respectively. Bivariate associations are again reported for descriptive purposes using Spearman correlations and between-group comparisons of AUC, intensity, and α were conducted using ANCOVAs with FTCD scores entered as a covariate, due to between-group differences in cigarette dependence (see below).

### 3.3. Results

#### 3.3.1. Sample Characteristics

One hundred and twenty-five participants failed at least one of the embedded attention checks and were omitted listwise. An additional six cases were omitted for providing incomplete episodic event information in the entry fields. Just 1 participant provided incomplete delay discounting data; 107 participants failed at least 1 of the criteria for systematic discounting (criterion 1 only: *n* = 32; criterion 2 only: *n* = 31; both criteria: *n* = 44); 14 participants failed at least one of the criteria for systematic demand (criterion 1 only: *n* = 6; criterion 2 only: *n* = 0; criterion 3 only: *n* = 6; multiple criteria (1 and 3): *n* = 2), 5 of which were among those also flagged for nonsystematic discounting data. This resulted in a final sample of *n* = 164 (EFT: *n* = 76; ERT: *n* = 88).

Participants did not significantly differ in age (*t*_162_ = 0.83, *p* = 0.41), sex (χ^2^_1, *N* = 164_ = 2.69, *p* = 0.10), race (χ^2^_1, *N* = 165_ = 0.02, *p* = 0.88), level of education (*U* = 3189, *p* = 0.60), income (*U* = 3328, *p* = 0.96), cigarettes smoked per day (*t*_162_ = 1.00, *p* = 0.32), or intention to quit smoking (*t*_162_ = 0.52, *p* = 0.60). Scores on the FTCD were higher for the ERT group (*m* = 4.74, *SD* = 1.71) than for the EFT group (*m* = 4.04, *SD* = 2.16; *t*_162_ = 2.32, *p* = 0.02) and were therefore included as a covariate in the general linear models, reported further below. Importantly, there were no significant differences in the degree to which participants were confident they had been assigned to the experimental condition (*t*_162_ = 1.64, *p* = 0.10), decreasing concerns that demand characteristics may have differentially influenced participant responding across the two groups. Group summary statistics are reported in Table 3.

#### 3.3.2. Bivariate Associations

Table 4 contains Spearman rank correlations between demographic, smoking, and behavioral economic estimates, a subset of which are reported here. Intensity of demand (i.e., consumption unconstrained by price) was highly and positively associated with cigarettes smoked per day (*r*_s_ = 0.70, *p* < 0.001) as well as scores on the FTCD (*r*_s_ = 0.50, *p* < 0.001). We observed the inverse for the α parameter, where lower values (indicating less sensitivity to increases in price) were moderately associated with cigarettes smoked per day (*r*_s_ = −0.33, *p* < 0.001) and scores on the FTCD (*r*_s_ = 0.21, *p* = 0.01). Participant age was positively associated with the number of cigarettes smoked per day (*r*_s_ = 0.29, *p* < 0.001) and inversely associated with confidence about having been assigned to the experimental condition (*r*_s_ = −0.17, *p* = 0.03). Importantly, there were no significant associations between study demand characteristics and delay discounting (*r*_s_ = 0.05, *p* = 0.50), intensity of demand (*r*_s_ = 0.00, *p* = 0.96), or the α parameter (*r*_s_ = −0.07, *p* = 0.41), further reducing concerns that our primary outcome variables varied as a function of participants’ belief that they were assigned to the experimental condition.

#### 3.3.3. Delay Discounting and Cigarette Demand

**Figure 3:**
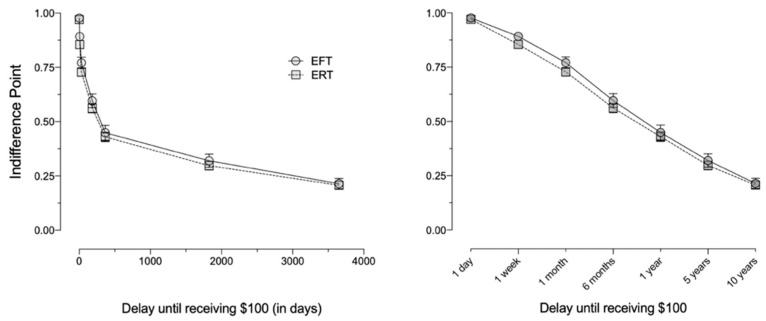
Mean indifference points (±*SEM*) on the delay discounting task for Experiment 2. EFT—episodic future thinking; ERT—episodic recent thinking. Left panel: delays presented on a linear *x*-axis to depict the shape of the discounting function. Right panel: delays presented on an *x*-axis with intervals equidistantly spaced to facilitate examination of all values.

**Figure 4:**
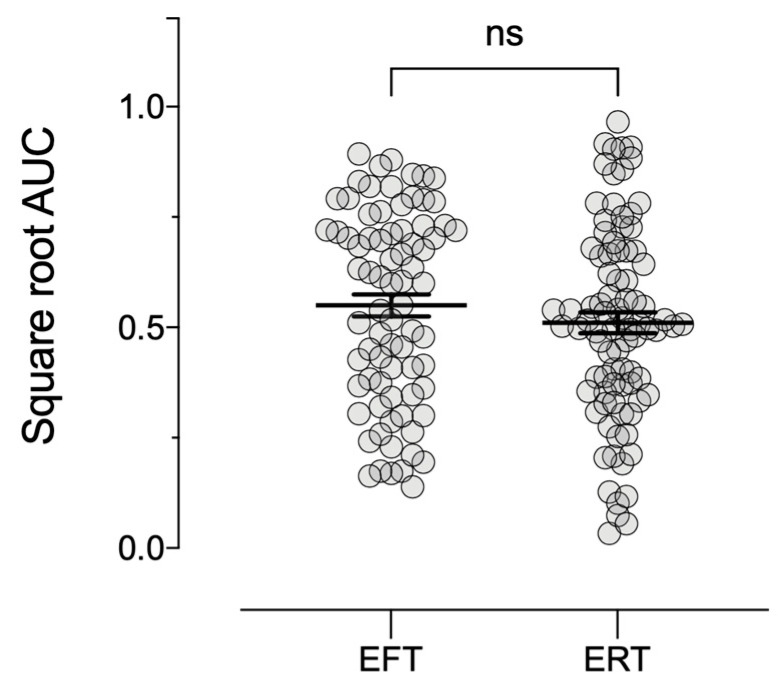
Mean square root transformed area under the curve (AUC) values (±*SEM*) for Experiment 2. EFT—episodic future thinking; ERT—episodic recent thinking.

Figure 3 and Figure 4 depict mean delay discounting indifference points and AUC, respectively, for the EFT and ERT groups. Delay discounting did not significantly differ as a function of FTCD scores (*F*_1,161_ = 0.00, *p* = 0.99, $\eta_{p}^{2}$ < 0.001) or episodic condition (*F* (1,161) = 1.03, *p* = 0.31, $\eta_{p}^{2}$ = 0.01).

**Figure 5:**
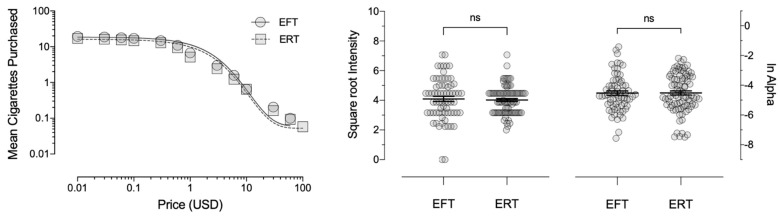
Left panel: demand curve for mean cigarettes purchased on the Cigarette Purchase Task in Experiment 2; Middle panel: mean square root transformed intensity values (±*SEM*); right panel: mean natural log transformed α values (±*SEM*). EFT—episodic future thinking; ERT—episodic recent thinking.

Hypothetical cigarette purchasing varied as an orderly function of increasing price (Figure 5), where Equation 1 described the mean consumption data well for the EFT (*R*^2^ = 0.96; *RMSE* = 0.16) and ERT (*R*^2^ = 0.97; *RMSE* = 0.17) groups. Results indicated a significant effect of FTCD scores on intensity (*F*_1,153_ = 51.81, *p* < 0.001, $\eta_{p}^{2}$ = 0.25); however, no significant differences emerged as a function of the episodic condition (*F*_1,153_ = 3.52, *p* = 0.06, $\eta_{p}^{2}$ = 0.02). Similarly, FTCD scores significantly predicted the α parameter (*F*_1,151_ = 10.56, *p* = 0.001, $\eta_{p}^{2}$ = 0.07), although there were no significant differences due to the episodic condition (*F*_1,151_ = 0.45, *p* = 0.51, $\eta_{p}^{2}$ = 0.003).

## 4. General Discussion

Results across the experiments reported here serve as call for further research into boundary conditions of previously reported behavioral economic findings. In Experiment 1, we employed a factorial design to compare the effects of EFT and GWLs on delay discounting and found no significant main effects of either manipulation. We note, however, that exposure to GWLs was associated with less discounting when paired with EFT and greater discounting when paired with ERT, evidenced by the significant EFT × GWL interaction. Future researchers may wish to clarify the conditions under which the combination of these two techniques can produce reliable differences in delay discounting among cigarette smokers. To better isolate the effects of EFT in Experiment 2, we recruited a new sample of smokers and assessed delay discounting and demand for cigarettes between EFT and ERT conditions without the use of GWLs. Results indicated no significant differences in discounting or demand as a function of EFT.

Given successful previously published demonstrations of the effects of EFT, we offer several hypotheses that may partially account for the discrepant findings. First, although the least discounting in Experiment 1 occurred when EFT was paired with GWLs, it remains plausible that the graphic images on the cigarette packs may have attenuated the effect of EFT. Indeed, evidence suggests that negative valence of imagined future events may temper the beneficial effects of EFT on delay discounting [44,45,46]. Although participants in the EFT conditions were instructed to imagine positive future events, it bears to reason that viewing images of acutely diseased lungs that could likely result as a future consequence of smoking may have partially counteracted the strength of the EFT manipulation. It is also possible that the EFT manipulations may have been affected by subtle procedural differences. Although the delay discounting task used in the present experiments had been previously validated [34] across dimensions including the shape of the discounting function (primarily hyperbolic), reward magnitude (small rewards were discounted more than large rewards), and reward domain (cigarettes were discounted more than money), the strength of the EFT manipulation may have been tempered by properties specific to the task. For example, asking participants to use a VAS to estimate the amount of money that would make them feel just as good at that moment as receiving a larger amount after a delay (i.e., to estimate their own indifference points) may have interfered with their ability to attend to their personally relevant future events. It is possible that these task properties may have produced similar tempering effects in the GWL conditions in Experiment 1.

Although the possibility of negative valence and task interference may provide a potential explanation for the null effects observed in the delay discounting assessments, it does not explain the absence of an effect on the CPT, where the EFT manipulation produced no differences in cigarette demand. It is possible that results similar to those reported here may exist in unpublished datasets, resulting in a “file drawer” effect that would indirectly inflate the robustness of these manipulations by assuring that only statistically significant demonstrations of these effects advanced to publication (cf. [28]). Importantly, if the effect of EFT is as sensitive to subtle procedural characteristics as the present experiments suggest, it raises the question as to whether the effect could hold under the far more complex conditions where decisions to smoke (or engage in other unhealthy behaviors) occur. Further research on the boundary conditions of EFT and other techniques aimed at reducing tobacco use is paramount. For example, evidence suggests that EFT can have muted effects on demand for alcohol [47] and may not be effective with older adults [48,49].

The findings of these experiments must also be considered within the context of their limitations. Although we applied best practices for ensuring mTurk data quality [50,51], a substantial proportion of the sample were flagged and omitted for failing embedded attention checks or providing nonsystematic data. In laboratory settings, research staff are better able to address participant misunderstandings as well as inattentive or rushed responding in real time; however, in online surveys, such cases can only be identified after survey completion and often result in removal. Relatedly, although there have been numerous successful demonstrations of EFT and GWL manipulations using online crowdsourced samples [27,52,53], it nevertheless bears to reason that the degree to which participants could imagine and think about their personally relevant future events and/or attend to the graphic images on the cigarette packs, may have been dependent on whether they were completing the tasks in environments with minimal distractions. This concern, however, was generally allayed by the fact that all participants were required to provide detailed descriptions of the episodic events as well as pass the embedded attention checks. We acknowledge, as a final limitation, that although previous research suggests significant overlap between hypothetical and experienced outcomes in behavioral economic tasks [15,16,54], the sole reliance on hypothetical outcomes in the present experiments limits the degree to which these findings may generalize.

## 5. Conclusions

Taken together, these results suggest that incorporating techniques like EFT as therapeutic smoking cessation interventions may require additional research, as the effects of these manipulations might be transient or impacted by subtle procedural differences. The authors encourage other researchers to disseminate their null results which could thereby help their respective fields avoid the pitfalls of the “file drawer” and identify boundary conditions that, if addressed, could increase the robustness of techniques like EFT.

## Figures and Tables

**Table 1:** Experiment 1 demographic characteristics (*N* = 105).

Variable	Experiment 1 Groups			
	Episodic Future Thinking		Episodic Recent Thinking	
	Regular Packs (*n* = 25)	Graphic Warning (*n* = 24)	Regular Packs (*n* = 28)	Graphic Warning (*n* = 28)
Sex (*n,* % female)	9 (36.00)	12 (50.00)	14 (50.00)	12 (42.86)
Age (*m* ± *SD*)	33.76 (10.71)	37.88 (11.04)	34.79 (9.10)	38.96 (10.54)
Race/ethnicity (*n*, %)				
White	19 (76.00)	23 (95.83)	25 (89.29)	24 (85.71)
Black	3 (12.00)	0	1 (3.57)	1 (3.57)
Asian	1 (4.00)	0	0	1 (3.57)
Hispanic/Latino	0	1 (4.17)	1 (3.57)	1 (3.57)
Native American/Alaskan Native	1 (4.00)	0	0	0
More than one	1 (4.00)	0	1 (3.57)	0
Other	0	0	0	1 (3.57)
Education				
Bachelor’s degree or higher (*n*, %)	9 (36.00)	11 (45.83)	11 (39.29)	13 (46.43)
Annual income ($, %)				
<10,000	1 (4.00)	3 (12.50)	4 (14.29)	0
10,000–19,000	2 (8.00)	4 (16.67)	2 (7.14)	2 (7.14)
20,000–29,000	5 (20.00)	1 (4.17)	2 (7.14)	2 (7.14)
30,000–39,000	4 (16.00)	3 (12.50)	3 (10.71)	4 (14.29)
40,000–49,000	2 (8.00)	2 (8.33)	3 (10.71)	4 (14.29)
50,000–59,000	4 (16.00)	4 (16.67)	3 (10.71)	8 (28.57)
60,000–69,000	2 (8.00)	4 (16.67)	3 (10.71)	2 (7.14)
70,000–79,000	1 (4.00)	1 (4.17)	1 (3.57)	1 (3.57)
80,000–89,000	2 (8.00)	2 (8.33)	3 (10.71)	1 (3.57)
90,000–99,000	0	0	1 (3.57)	2 (7.14)
100,000–149,000	1 (4.00)	0	3 (10.71)	2 (7.14)
>150,000	1 (4.00)	0	0	0
Cigarettes per day (*m* ± *SD*)	11.36 (6.45)	14.21 (9.08)	16.11 (10.58)	16.11 (9.19)
FTCD (*m* ± *SD*)	3.92 (1.98)	4.79 (2.25)	4.75 (2.24)	4.75 (2.03)
Contemplation Ladder (*m* ± *SD*)	6.36 (2.94)	5.29 (3.11)	6.18 (2.31)	5.89 (2.36)

Note. FTCD—Fagerström Test for Cigarette Dependence.

**Table 2:** Spearman rank correlations among demographic characteristics, smoking, and delay discounting.

Variable	1	2	3	4	5	6
1. Age						
2. Education	−0.03					
3. Income	0.09	0.26 **				
4. Cigarettes/day	0.40 **	−0.26 **	−0.07			
5. FTCD	0.27 **	−0.22 *	−0.05	0.72 **		
6. Contemplation Ladder	0.07	−0.02	−0.01	−0.09	−0.26 **	
7. AUC	0.15	0.13	0.21 *	−0.01	−0.09	−0.06

Note. FTCD—Fagerström Test for Cigarette Dependence; AUC—area under the discounting curve; * *p* < 0.05; ** *p* < 0.01.

**Table 3:** Experiment 2 Demographic Characteristics (*n* = 164).

Variable	Experiment 2 Groups	
	Episodic Future Thinking (*n* = 76)	Episodic Recent Thinking (*n* = 88)
Sex (*n,* % female)	30 (39.47)	46 (52.27)
Age (*m* ± *SD*)	36.30 (9.93)	34.94 (8.92)
Race/ethnicity		
White	62 (81.58)	70 (79.55)
Black	4 (5.26)	5 (5.68)
Asian	5 (6.58)	2 (2.27)
Hispanic/Latino	1 (1.32)	5 (5.68)
Native American/Alaskan Native	2 (2.63)	1 (1.14)
More than one	2 (2.63)	4 (4.55)
Other	0	1 (1.14)
Education		
Bachelor’s degree or higher (*n*, %)	29 (38.16)	36 (40.91)
Annual income ($, %)		
<10,000	6 (7.89)	8 (9.09)
10,000–19,000	7 (9.21)	5 (5.68)
20,000–29,000	9 (11.84)	10 (11.36)
30,000–39,000	10 (13.16)	15 (17.05)
40,000–49,000	14 (18.42)	19 (21.59)
50,000–59,000	8 (10.53)	8 (9.09)
60,000–69,000	9 (11.84)	8 (9.09)
70,000–79,000	6 (7.89)	3 (3.41)
80,000–89,000	1 (1.32)	2 (2.27)
90,000–99,000	1 (1.32)	3 (3.41)
100,000–149,000	4 (5.26)	4 (4.55)
>150,000	1 (1.32)	3 (3.41)
Cigarettes per day (*m* ± *SD*)	13.72 (6.75)	12.63 (6.02)
FTCD (*m* ± *SD*)	4.04 (2.16)	4.74 (1.71) *
Contemplation Ladder (*m* ± *SD*)	6.26 (2.59)	6.06 (2.48)

Note. FTCD—Fagerström Test for Cigarette Dependence. * *p* < 0.05. See text for statistical comparisons.

**Table 4:** Spearman rank correlations among demographic characteristics, smoking, and behavioral economic variables.

Variable	1	2	3	4	5	6	7	8	9
1. Age									
2. Education	0.04								
3. Income	0.21 **	0.31 **							
4. Cigarettes/day	0.29 **	−0.11	0.02						
5. FTCD	0.12	−0.16 *	0.01	0.57 **					
6. Contemplation Ladder	0.10	−0.01	0.08	−0.13	−0.08				
7. AUC	0.00	0.13	0.11	0.03	−0.04	−0.03			
8. Intensity	0.08	−0.11	−0.07	0.70 **	0.50 **	−0.13	−0.01		
9. α	0.06	−0.11	−0.14	−0.33 **	−0.21 **	0.20 *	−0.13	−0.35 **	
10. Demand characteristics	−0.17 *	0.02	0.05	−0.04	0.06	−0.06	0.05	0.00	−0.07

Note. FTCD—Fagerström Test for Cigarette Dependence; AUC—area under the discounting curve; Demand characteristics—the degree to which participants rated they were confident that they had been assigned to the experimental group. * *p* < 0.05; ** *p* < 0.01.

## Data Availability

Data are available upon request from the corresponding author.

## Supplementary Materials

The following are available online at https://www.mdpi.com/article/10.3390/ijerph182312637/s1, Example instructions for episodic future thinking conditions.
