# Supplementary material for: The Influence of Episodic Future Thinking and Graphic Warning Labels on Delay Discounting and Cigarette Demand
Source: Int J Environ Res Public Health. 2021 Nov 30;18(23):12637. doi: 10.3390/ijerph182312637 (PMC8656814; doi:10.3390/ijerph182312637)
Supplement: Supplementary file 1 [file ijerph-18-12637-s001.zip › ijerph-1420529-supplementary.pdf]

**Supplemental Materials**

**Example instructions for episodic future thinking conditions (cigarette pack[1] and graphic warning in Experiment 1 only)**

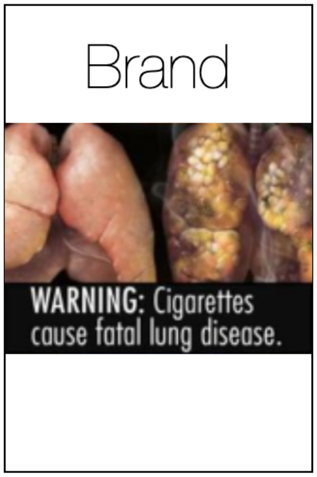


Please imagine a realistic, positive event that you are looking forward to occurring in **1 year**. Try to envision a specific event as vividly as possible, as you will be asked to describe the event on the following page. Imagine where you will be, whom you will be with, what you will be doing, and any sensations (sights, sounds, smells, etc.) associated with the event. The event should not be specifically about smoking. To encourage vivid imagining, **you will not be able to access the next question until 1 minute has elapsed.**

---

In this imagined, future event, where will you be?

In this imagined, future event, what will you be doing?

In this imagined, future event, who will be with you?

---

[1] The cigarette pack displayed was of the participants' preferred brand (selected at the beginning of the experiment). Participants assigned to one of the regular pack conditions viewed an image of their preferred pack without the graphic warning label.

In this imagined, future event, how will you feel?

Please provide a one-sentence summary of the imagined event. Please include any relevant details such as where you will be, what you will be doing, etc.

**Example instructions for episodic recent thinking conditions (cigarette pack and graphic warning in Experiment 1 only)**

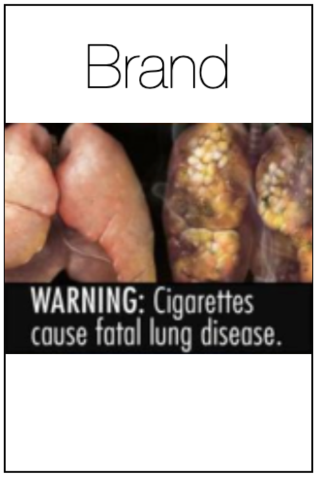


Please remember a positive event that occurred in your life **1 day ago**. Try to envision a specific event as vividly as possible, as you will be asked to describe the event on the following page. Visualize where you were, whom you were with, what you were doing, and any sensations (sights, sounds, smells, etc.) associated with the event. The event should not be specifically about smoking. To encourage vivid remembering, **you will not be able to access the next question until 1 minute has elapsed**.

Briefly (one to two sentences), where were you in the remembered event?

Briefly (one to two sentences), whom were you with in the remembered event?

Briefly (one to two sentences), how did you feel during the remembered event?

Please provide a one-sentence summary of the remembered event. Please include any relevant details such as where you were, what you were doing, etc.

---

**Delay discounting task instructions**[2]

**For each of the next 8 questions, imagine the following hypothetical (pretend) scenario:**

You are presented with a choice between money now or later.

For the money now option, the money is deposited automatically into your bank account immediately.

For the money later option, the money is automatically deposited into your bank account after the specified time.

Your job is to use the slider tool to tell us the amount of money that you would like to receive immediately that would make you feel JUST AS GOOD as you would if you were to receive money after the specified time.

**Delay discounting task instructions (continued)**

Although the scenarios are pretend, we ask that you consider each scenario as if it was real and as if it was the only scenario you would face today. Finally, when considering each scenario, you should take into account your financial circumstances (e.g., current account balance, rent or bills due).

---

[2] Delay discounting was assessed across 8 questions, one of which served as an embedded attention check ("Please slide the slider to 0 to indicate you are paying attention") and was not included in the analysis.

**Example episodic future thinking discounting trial**

***I will be on vacation in Paris visiting the Louvre***

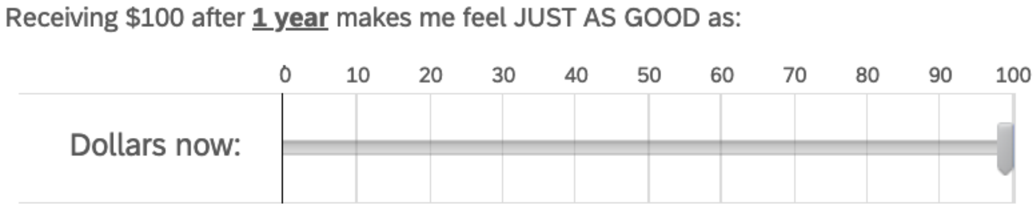


---

**Example episodic recent thinking discounting trial**

***I was at home working in the garden***

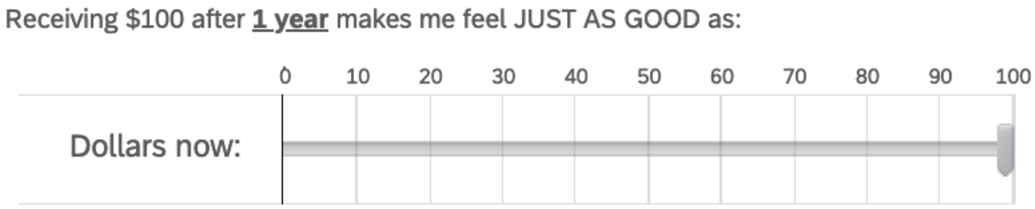


---

**Cigarette purchase task instructions**[3]

The following questions are hypothetical (pretend), but please answer as though the consequences were real. This means you should take into account your current financial situation and any other factors about your current life circumstances when answering.

Imagine that you have finished the study and will spend the next day in your usual home environment. Also imagine that you have the chance to buy your usual brand of cigarettes for your own personal use within the next day (24 hours). You can buy as many cigarettes as you like, but you cannot sell, trade, or give them away, and you cannot save them for more than a day. Other than the fact that the cigarettes are for your own use within the next day, there is no limit to the number of cigarettes you can buy. Please do not buy more than you will use.

[3] Cigarette demand was assessed across 14 questions, one of which served as an embedded attention check ("Please type "1812" in the box below to indicate you are paying attention") and was not included in the analysis.

The following questions will ask you how many of your usual brand of cigarettes you would buy if they were sold at various prices. For each question, enter the number of cigarettes you would buy, and enter zero if you wouldn't buy any at that price. Please consider each of the questions separately, meaning that if you buy cigarettes in one question, pretend that you don't have them when you answer the other questions. In other words, when you are answering each question, pretend that it is the only question being asked of you today.

---

**Example episodic future thinking purchase task trial**

Reminder:

- The questions ask how many of your usual brand of cigarettes you would buy to use over the next day (24 hours).
- You can't sell/trade/give away any of the of the cigarettes that you buy.
- The cigarettes that you buy are for your own use in the next day.
- This is the only place that you can buy cigarettes in the next day.
- Do not buy more cigarettes than you will use in the next day.
- Answer each question as if it's the only question being asked of you today (if you buy cigarettes during one question, pretend that you don't have them when you answer the other questions).
- If you would not buy any cigarettes at a certain price, please enter a '0' in the box.

Before answering, please take a moment to think about the scenario you described previously, then respond with how many cigarettes you would purchase in the next 24 hours:

***I will be backpacking through Europe with my partner***

If the cigarettes cost **1 dollar** each,
How many cigarettes would you buy to use in the next 24 hours?

**Example episodic recent thinking purchase task trial**

Reminder: future

- The questions ask how many of your usual brand of cigarettes you would buy to use over the next day (24 hours).
- You can't sell/trade/give away any of the of the cigarettes that you buy.
- The cigarettes that you buy are for your own use in the next day.
- This is the only place that you can buy cigarettes in the next day.
- Do not buy more cigarettes than you will use in the next day.
- Answer each question as if it's the only question being asked of you today (if you buy cigarettes during one question, pretend that you don't have them when you answer the other questions).
- If you would not buy any cigarettes at a certain price, please enter a '0' in the box.

Before answering, please take a moment to think about the scenario you described previously, then respond with how many cigarettes you would purchase in the next 24 hours:

***I was having dinner at my partner's house***

If the cigarettes cost **1 dollar** each,
How many cigarettes would you buy to use in the next 24 hours?
